# Switching off Bacterial Flagellar Biogenesis by YdiU-Mediated UMPylation of FlhDC

**DOI:** 10.1128/mbio.00249-22

**Published:** 2022-05-09

**Authors:** Yue Ma, Yingying Yue, Haihong Jia, Nannan Song, Li Zhai, Weiwei Wang, Cuiling Li, Bingqing Li

**Affiliations:** a Department of Clinical Laboratory, Shandong Provincial Hospital Affiliated to Shandong First Medical University, Jinan, Shandong, China; b Department of Pathogen Biology, School of Basic Medicine, Shandong First Medical University & Shandong Academy of Medical Sciences, Jinan, China; c Key Lab for Biotech-Drugs of National Health Commission, Jinan, Shandong, China; d Key Lab for Rare & Uncommon Diseases of Shandong Province, Jinan, Shandong, China; University of California, Berkeley

**Keywords:** FlhDC, *Salmonella*, UMPylation, YdiU, flagellar gene regulation

## Abstract

Bacterial flagellin activates the host immune system and triggers pyroptosis. Salmonella reduces flagellin expression when it survives within host cells. Here, we found that the UMPylator YdiU significantly altered the Salmonella flagellar biogenesis process upon host cell entry. The expression levels of class II and class III flagellar genes, but not the class I flagellar genes *flhDC*, were dramatically increased in a Δ*ydiU* strain compared to wild-type (WT) Salmonella in a host-simulating environment. A direct interaction between YdiU and FlhDC was detected by bacterial two-hybrid assay. Furthermore, YdiU efficiently catalyzed the UMPylation of FlhC but not FlhD, FliA, or FliC. UMPylation of FlhC completely eliminated its DNA-binding activity. *In vivo* experiments showed that YdiU was required and sufficient for Salmonella flagellar control within host cells. Mice infected with the Δ*ydiU* strain died much earlier than WT strain-infected mice and developed much more severe inflammation and injury in organs and much higher levels of cytokines in blood, demonstrating that early host death induced by the Δ*ydiU* strain is probably due to excessive inflammation. Our results indicate that YdiU acts as an essential factor of Salmonella to mediate host immune escape.

## INTRODUCTION

Salmonella is a major pathogen, causing food poisoning in humans and animals and other serious diseases, such as typhoid fever (1, 2). Bacterial flagella allow motility and are required for Salmonella to successfully invade host cells (3–5). However, flagellin can be recognized by host receptors, such as Toll-like receptor 5 (TLR5) and Ipaf, which then activate the host immune response to reduce Salmonella survival in host cells (6, 7). The expression of the major flagellar antigen FliC quickly decreases more than 10-fold upon wild-type Salmonella entry into host cells (8, 9). Remarkably, Salmonella organisms continuously expressing FliC exhibit weaker pathogenicity and elicit stronger host immune responses (10–12), suggesting that precise regulation of the flagellar pathway is critical for successful infection by Salmonella.

The synthesis and assembly of the Salmonella flagellum involves more than 70 genes on 25 operons (13, 14). The earliest-transcribed genes are *flhD* and *flhC*. The products FlhD and FlhC assemble into the FlhD_4_C_2_ complex, which binds to the promoter regions of the secondary flagellar genes and recruits RNA polymerase to initiate the synthesis of these genes (15, 16). Most regulation of flagellar synthesis targets *flhDC*, making *flhDC* the “master switch” of the flagellar synthesis pathway. The transcription of *flhDC* genes is regulated by CAP, H-NS, RcsB, RflM, HilD, and YjjQ factors (17–21). The translation of *flhDC* is controlled by CsrA (22). DnaK is required for correct folding of the FlhD_4_C_2_ complex (23). The FlhD_4_C_2_ complex can be degraded by the ClpXP protease (24, 25). Three other factors, FliT, YdiV, and STM1697, modulate FlhDC function through direct interactions with the FlhDC complex (26–28). However, no posttranslational modifications of FlhDC have been reported to regulate flagellar synthesis.

YdiU belongs to a widespread and highly conserved protein family and functions as a modifying enzyme that catalyzes posttranslational protein modifications (29, 30). YdiU catalyzes the UMPylation of multiple proteins in Salmonella (30). Preliminary screening identified UMPylation of 46 proteins in Salmonella, suggesting that YdiU may help regulate Salmonella life processes through UMPylation (30). To further investigate the physiological functions of Salmonella YdiU, wild-type and YdiU knockout Salmonella strains were cultured in a host cell-simulating iron-deficient environment, and then genome-wide protein expression analysis was performed (45). The results showed that after YdiU deletion, genes related to flagellar synthesis and SPI-1 virulence in Salmonella exhibited significantly upregulated expression, while genes related to iron uptake, nitrogen metabolism, and carbon fixation exhibited significantly downregulated expression. The above data suggest that YdiU functions as a global regulator involved in multifarious metabolic activities of Salmonella.

In this study, we investigated the regulatory mechanism of flagellar synthesis by YdiU in Salmonella. We found that YdiU significantly inhibited the synthesis of Salmonella flagella under iron-deficient conditions. Interestingly, YdiU repressed the expression of secondary and tertiary flagellar genes but did not affect the transcription and expression of FlhDC. Bacterial two-hybrid assays demonstrated a direct interaction between YdiU and FlhDC, and YdiU was found to directly UMPylate the Ser31 residue of the FlhC in the FlhDC complex both *in vivo* and *in vitro*. The Ser31 residue is located on the DNA binding surface of FlhDC, suggesting that UMPylation could affect the DNA binding activity of FlhDC. Electrophoretic mobility shift assay (EMSA) experiments confirmed that the UMPylated FlhDC complex completely lost its DNA binding activity. Cellular infection experiments demonstrated that upon entry of wild-type Salmonella into host cells, YdiU effectively inhibited the expression of secondary and tertiary flagellar genes. The Δ*ydiU* strain could not turn off the expression of flagellar antigen upon entry into host cells. Animal experiments demonstrated that compared with wild-type Salmonella, infection with the Δ*ydiU* strain caused a more severe immune response and subsequent injury of organs, confirming that YdiU-mediated flagellar regulation is an important immune escape strategy of Salmonella within host cells. Thus, our results reveal a novel mechanism by which Salmonella adopts posttranslational modification to shut down flagellar synthesis as a strategy to achieve immune escape.

## RESULTS

### YdiU negatively regulates the flagellar synthesis pathway in Salmonella.

**FIG 1:**
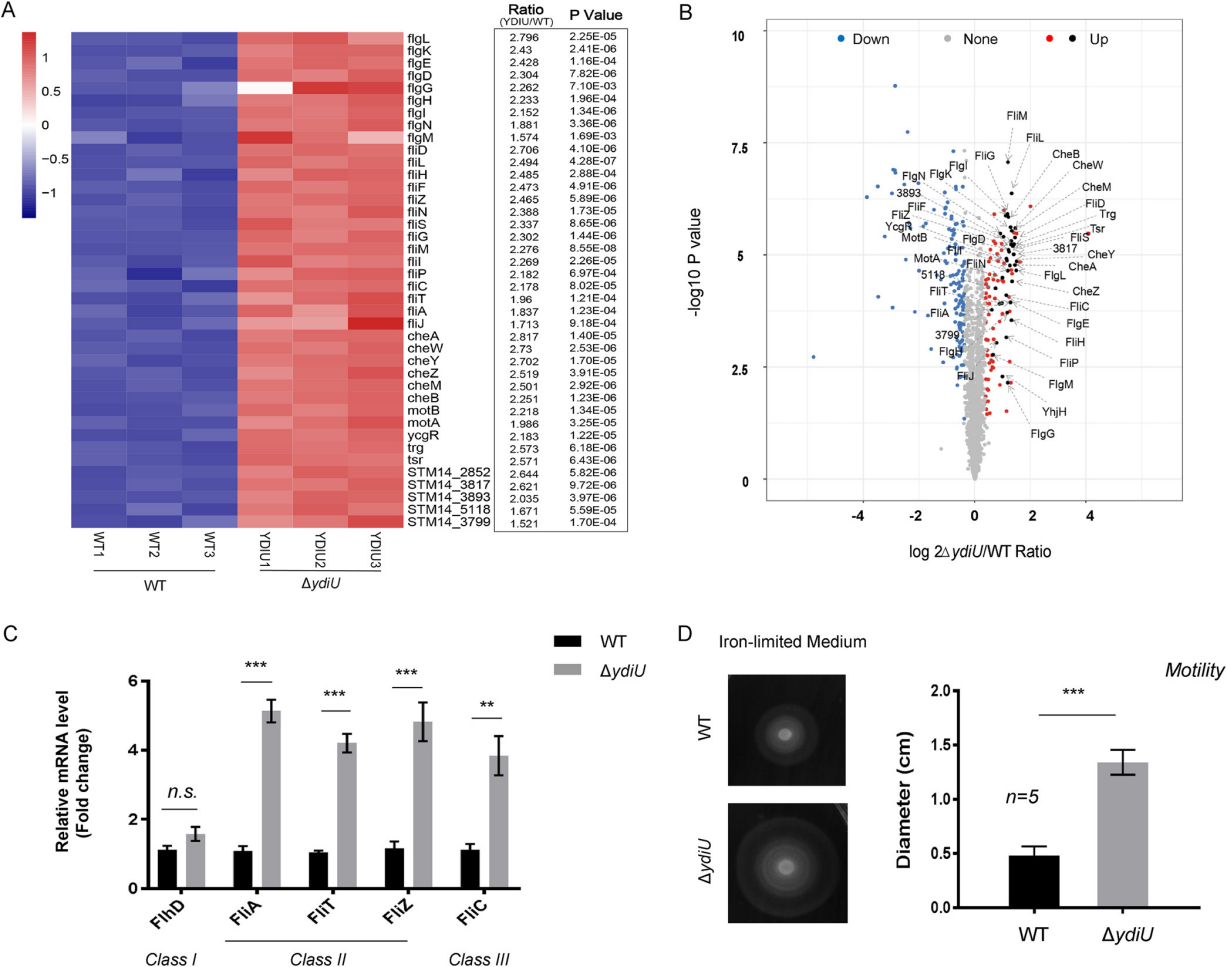
YdiU inhibits the flagellar synthesis pathway of Salmonella. (A and B) Differentially expressed flagellar proteins of WT and Δ*ydiU* under iron-limited conditions. (A) Heat map of the fold changes of flagellum-related proteins. (B) Total volcano plots of the proteomic results. The blue and red spots represent the downregulated and upregulated proteins, respectively. Flagellum-related proteins are labeled. (C) The mRNA levels of four flagellar genes in WT and Δ*ydiU* strains under iron-limited conditions were separately determined by qRT-PCR. (D) The motility behavior of WT and Δ*ydiU* strains was measured with soft-agar plates using iron-limited medium. The above-described experiments were performed as three replicates or five replicates (D), and means and standard errors of the means (SEM; error bars) are presented. ***, *P* < 0.001; **, *P* < 0.01; n.s., not significant (*P* > 0.05).

We showed in a previous study that YdiU was only expressed when Salmonella encountered stress signals (30). Genome-wide protein expression analysis with wild-type and Δ*ydiU* Salmonella demonstrated that YdiU has a close relationship with flagellar synthesis and bacterial chemotaxis (45). Further proteomic data analysis for flagellar pathway showed that 26 flagellum-associated proteins and 14 chemotaxis-associated proteins exhibited >2-fold-increased expression in the Δ*ydiU* strain compared with wild-type Salmonella, suggesting that YdiU may negatively regulate flagellar synthesis in Salmonella (Fig. 1A and B). To verify the proteomic results, we examined the expression of flagellar genes using quantitative real-time PCR (qRT-PCR) with wild-type and Δ*ydiU* Salmonella under iron-limited or iron-rich conditions. The mRNA level of the flagellar genes *fliA*, *fliT*, *fliZ*, and *fliC* increased 5.14-, 4.21-, 4.83-, and 3.84-fold in the Δ*ydiU* strain relative to that of the WT under iron-limited condition (Fig. 1C). However, these flagellar genes did not show differential expression in WT and Δ*ydiU* strains under iron-rich conditions (Fig. S1A). Moreover, there was no significant difference in the mRNA levels of class I flagellar *flhD* under iron-limited and iron-rich conditions (Fig. 1C and Fig. S1A). The motility behavior of WT and Δ*ydiU* strains was assayed using soft-agar plates. The Δ*ydiU* strain exhibited significantly enhanced motility compared to the wild-type strain under iron-limited conditions (Fig. 1D), while there was no significant difference between the strains under iron-rich conditions (Fig. S1B). These data suggest that YdiU negatively regulates motility under iron-limited conditions by inhibiting the expression of class II and class III flagellar genes.

10.1128/mbio.00249-22.1 FIG S1 YdiU did not inhibit flagellar biogenesis under conventional conditions. (A) The mRNA levels of four flagellar genes in WT and Δ*ydiU* strains in LB medium were separately determined by qRT-PCR. (B) The motility behaviors of WT and Δ*ydiU* strains were measured with soft-agar plates using LB medium. The above-described experiments were performed as three replicates (A) or five replicates (B), and the mean values are presented. **, *P* < 0.01; n.s., *P* > 0.05. Download FIG S1, TIF file, 0.4 MB. Copyright © 2022 Ma et al. 2022 Ma et al. https://creativecommons.org/licenses/by/4.0/ This content is distributed under the terms of the Creative Commons Attribution 4.0 International license.

### Regulation of flagellar synthesis by YdiU depended on its UMPylation activity.

**FIG 2:**
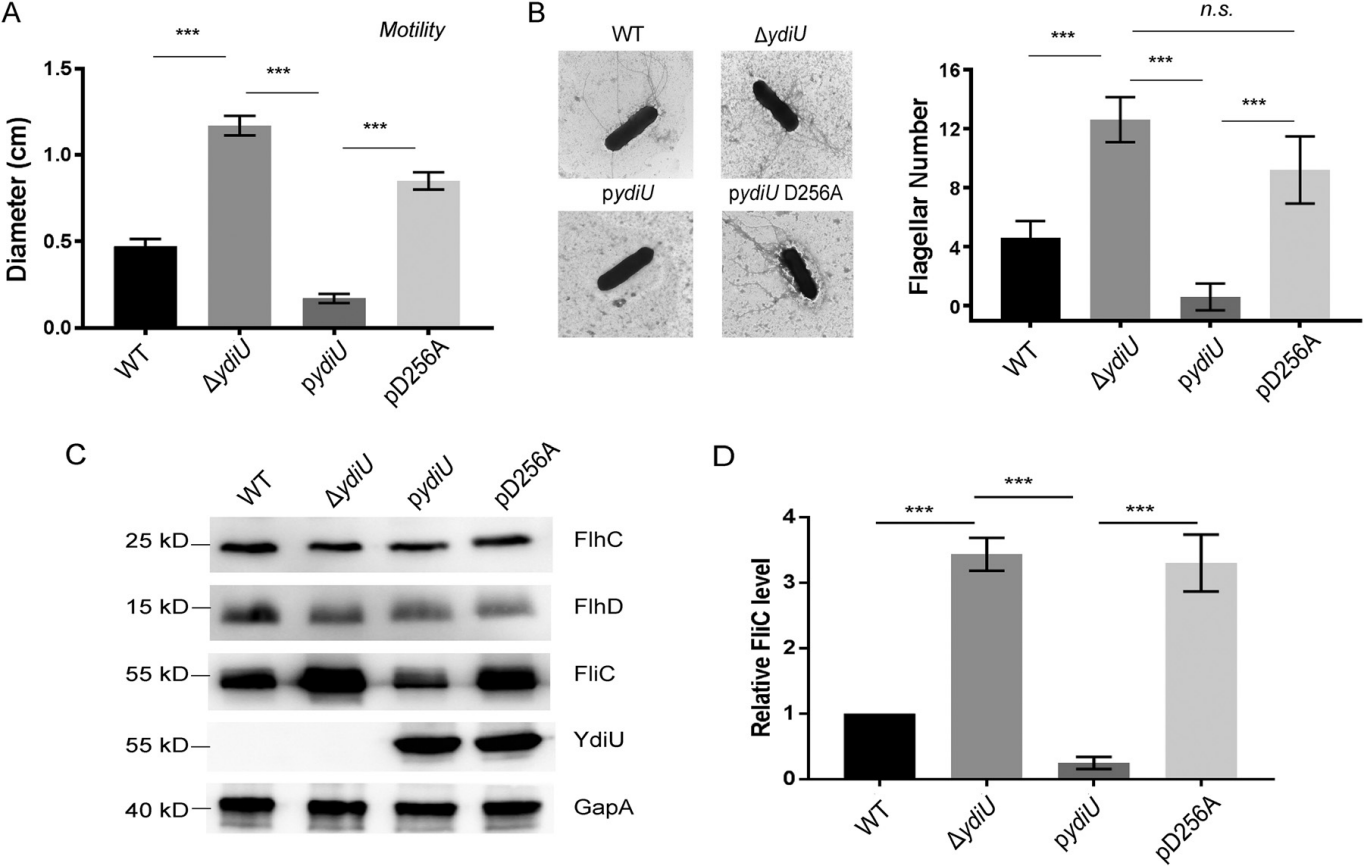
YdiU inhibits the expression of FliC in an UMPylation activity-dependent manner. (A) The motility behavior of four Salmonella strains was measured with soft-agar plates using iron-limited medium. (B) The numbers of flagella of the strains were observed using negative-staining electron microscopy (EM). The above-described experiments were performed as five replicates, and the means and SEM are presented. ***, *P* < 0.001; n.s., *P* > 0.05. (C) The protein levels of FlhD, FlhC, FliC, and YdiU were determined by Western blotting. GapA was used as a loading control. (D) The FliC levels relative to that of GapA were quantified by grayscale in three independent experiments. The gray values were obtained using ImageJ.

To further investigate the relationship between YdiU expression and bacterial motility, four Salmonella strains were tested: the wild type, the Δ*ydiU* strain, and Δ*ydiU* strains overexpressing wild-type YdiU or YdiU D256A lacking UMPylation activity (Fig. S2). The motility phenotypes of these strains were compared, and the results showed that the strain overexpressing YdiU barely moved on iron-limited soft agar, further demonstrating that YdiU could inhibit bacterial motility (Fig. 2A). The strain expressing the YdiU D256A mutant did not exhibit inhibition of motility, so we hypothesize that the YdiU-mediated inhibition of bacterial motility requires its enzymatic activity. Given that bacterial motility is associated with multiple pathways, such as flagellar synthesis, chemotaxis, and energy supply, we next determined the growth of flagellar filaments of these strains using negative-staining electron microscopy (Fig. 2B). More flagella were observed in the Δ*ydiU* strain than in the wild-type strain, with fewer flagella observed in Salmonella overexpressing YdiU (p*ydiU*) than in Salmonella overexpressing YdiU D256A (pD256A; the flagellar phenotype of the strain containing pD256A was similar to that of the Δ*ydiU* strain). Overall, the data demonstrate that YdiU is a negative flagellar regulator and that its inhibitory effect on flagellar growth depends on its enzymatic activity.

10.1128/mbio.00249-22.2 FIG S2 Expression of YdiU in strains used in this study. Global protein expression was detected by SDS-PAGE with the four strains used for Fig. 2. The overexpressed bands of YdiU (55 kDa) are highlighted. Download FIG S2, TIF file, 1.2 MB. Copyright © 2022 Ma et al. 2022 Ma et al. https://creativecommons.org/licenses/by/4.0/ This content is distributed under the terms of the Creative Commons Attribution 4.0 International license.

### Regulation of flagellum by YdiU occurs after *flhDC* transcription.

Proteomic analysis revealed that class II and class III flagellar proteins were significantly upregulated after the deletion of the *ydiU* gene, but there was no significant change in the amount of class I flagellar protein FlhDC, the most critical regulatory protein in the flagellar synthesis pathway. This finding of an unchanged protein level was consistent with qRT-PCR experiments that showed no change in the transcription of the primary flagellar gene *flhD* (Fig. 1C). To further investigate the role of YdiU, we investigated the levels of YdiU, FlhDC, and FliC flagellar proteins in the above-described four strains by Western blotting (Fig. 2C). The results showed that the expression of YdiU barely affected the level of FlhD and FlhC proteins but significantly reduced the amount of FliC (Fig. 2D). These results suggest that the regulatory effect of YdiU on flagellar biogenesis occurs mainly after the transcription of the dominant flagellum manipulator FlhDC. The expression of YdiU D256A exhibited no inhibitory effect on FlhDC, further confirming the pivotal role of enzymatic activity on YdiU-mediated flagellar control.

### YdiU UMPylates the FlhC subunit of FlhDC.

**FIG 3:**
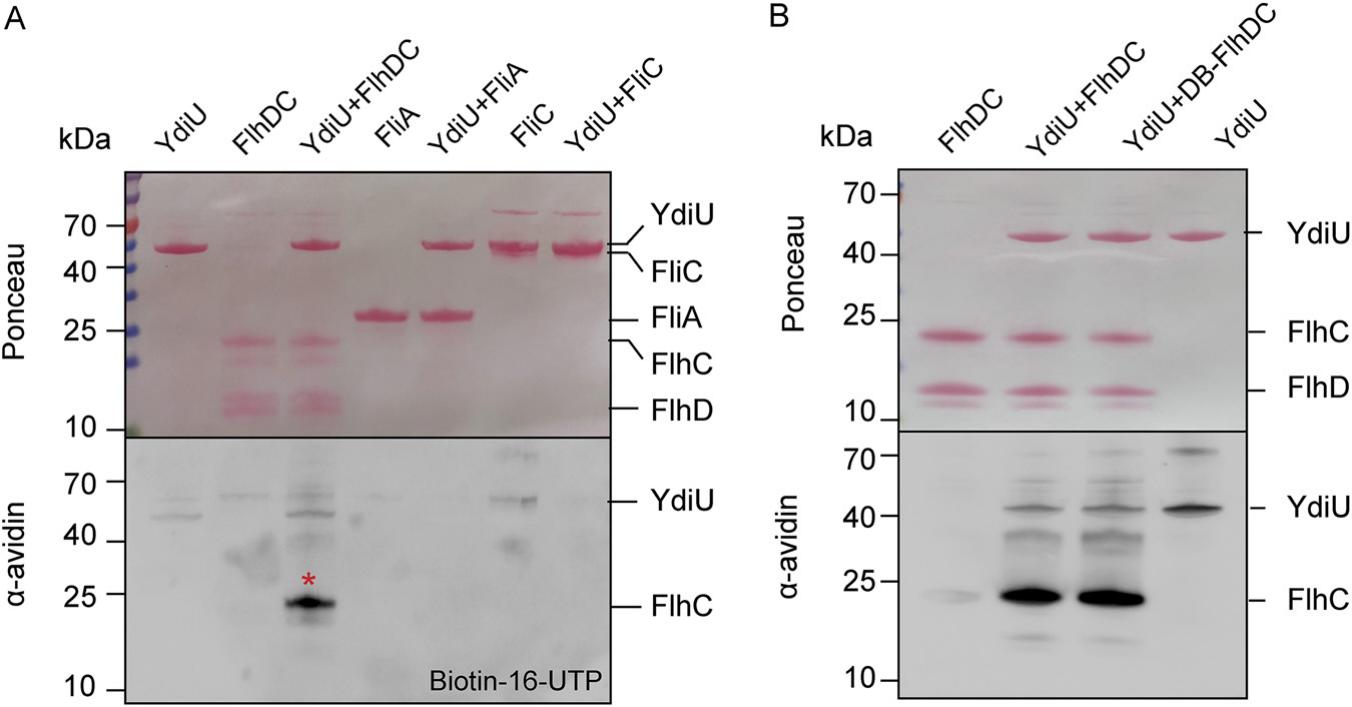
YdiU UMPylates the FlhC subunit of FlhDC. (A) *In vitro* UMPylation assays of FlhDC, FliA, and FliC by YdiU were separately performed with biotin-16-UTP. The UMPylated proteins were detected by streptavidin-HRP blotting, and total proteins were visualized by Ponceau S staining. (B) DNA binding does not inhibit the *in vitro* UMPylation of FlhC by YdiU. The DNA-bound FlhDC (DB-FlhDC) was prepared by incubation with excess target DNA for 10 min and used for *in vitro* UMPylation assays. The UMPylated proteins were detected by streptavidin-HRP blotting, and total proteins were visualized by Ponceau S staining.

Because YdiU inhibits flagellar synthesis in Salmonella without affecting the transcription and expression of FlhDC, we hypothesized that YdiU may act directly on the FlhDC protein. Therefore, we investigated the interaction between YdiU and FlhDC using a bacterial two-hybrid approach. The experimental results indicated a weak interaction of YdiU with FlhDC (Fig. S3). We previously reported that YdiU is a posttranslational modifying enzyme that UMPylates proteins (30), so we speculated that YdiU may function as a direct UMPylator of flagellar proteins. To test our hypothesis, the key flagellar proteins FlhDC, FliA, and FliC were purified and *in vitro* UMPylation experiments were performed using purified YdiU. YdiU was able to catalyze the UMPylation of FlhC, but not that of FlhD, FliA, and FliC (Fig. 3A). To probe the effect of DNA binding on the UMPylation of FlhDC, we incubated FlhDC with excess target DNA for 10 min and then performed the *in vitro* UMPylation assay. The results showed that the presence of excess DNA did not prevent UMPylation of FlhC (Fig. 3B).

10.1128/mbio.00249-22.3 FIG S3 Direct interaction between YdiU and FlhDC was determined by the bacterial two-hybrid method. (A) LB–X-Gal plates. PC, positive control (recombinant strain BTH101 containing T18-zip and T25-zip vectors); NC, negative control (recombinant strain BTH101 containing the empty T18 and T25 vectors) (B) Corresponding strains were tested by β-galactosidase assays. The values represent the means and standard deviations of three repeated results. ***, *P* < 0.001 compared with the negative control using a *t* test. Download FIG S3, TIF file, 2.1 MB. Copyright © 2022 Ma et al. 2022 Ma et al. https://creativecommons.org/licenses/by/4.0/ This content is distributed under the terms of the Creative Commons Attribution 4.0 International license.

**FIG 4:**
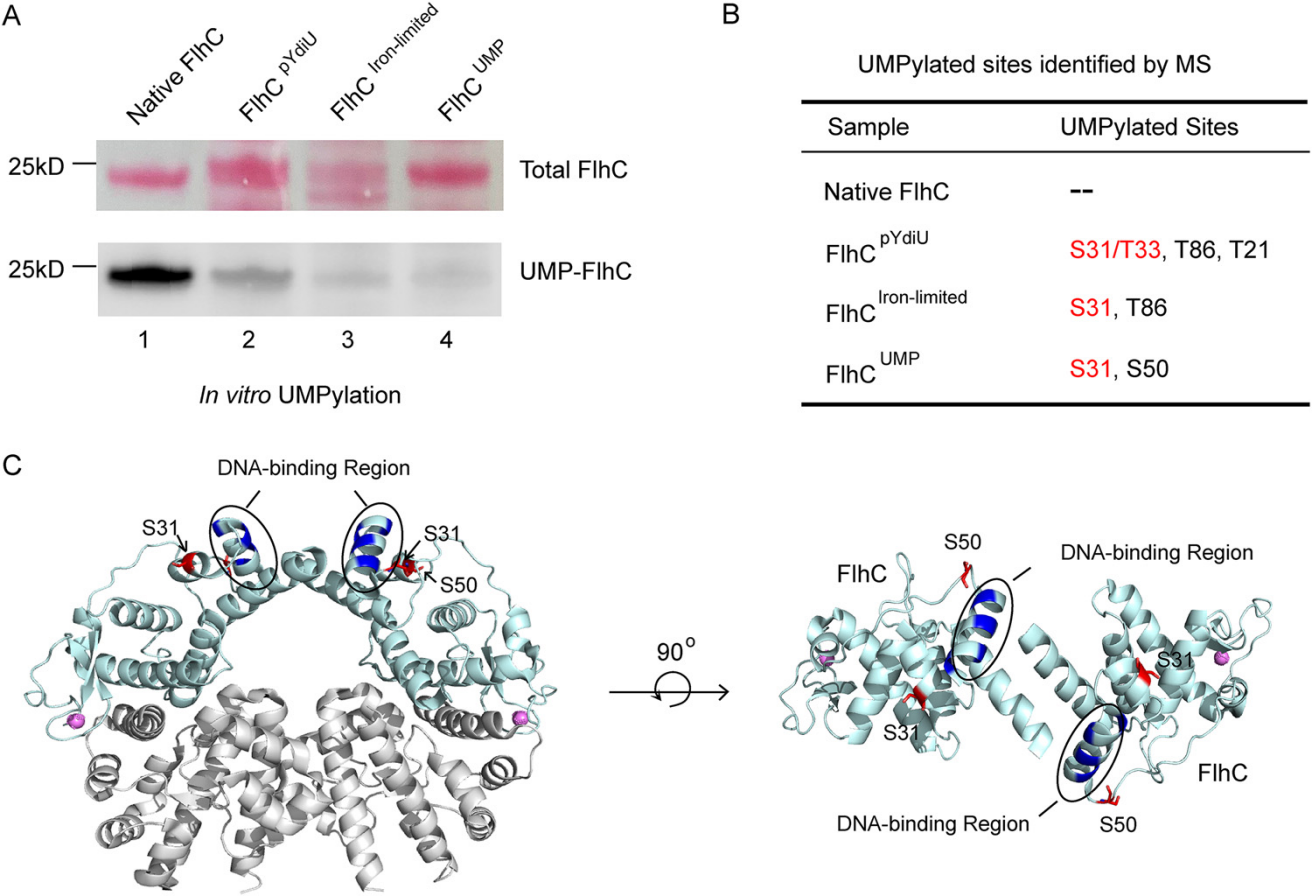
The UMPylated sites of FlhC are located at the DNA-binding region. (A) *In vitro* UMPylation assays of the four FlhDC proteins were separately performed with biotin-16-UTP. The UMPylated proteins were detected by streptavidin HRP blotting, and total proteins were visualized by Ponceau S staining. (B) UMPylation sites of FlhC were identified by mass spectrometry in the four FlhDC proteins. (C) Structural presentation showing that S31 and S50 of FlhC are located in the DNA binding region.

To map the site of modification, we prepared four kinds of FlhDC proteins: (i) native FlhDC purified from Escherichia coli with no source of YdiU (native FlhDC), (ii) FlhDC purified from YdiU-expressing E. coli (FlhDC^pYdiU^), (iii) FlhDC purified from E. coli cultured under iron-limited conditions (FlhDC^iron-limited^), and (iv) purified FlhDC UMPylated by YdiU *in vitro* (FlhDC^UMP^). Assays for YdiU-catalyzed UMPylation were performed using these four FlhDC protein variants and biotin-16-UTP (Fig. 4A). Native FlhDC was efficiently biotin-UMPylated by YdiU; however, the other three purified FlhDC proteins were not efficiently biotin-UMPylated, suggesting that those FlhDC proteins might already be UMPylated. To test this, the four FlhDC variants were analyzed by mass spectrometry to assess UMP modifications (Fig. 4B). The results were as follows: (i) no UMP modification was detected on the native FlhDC; (ii) UMPylation was detected on S31, T33, T86, and T21 residues in FlhDC^pYdiU^; (iii) UMPylation was detected on S31 and T86 in FlhDC^iron-limited^; and (iv) UMPylation was detected on S31 and S50 in FlhDC^UMP^.

To further determine the importance of UMPylation on above sites, we constructed six mutants of FlhC in the coexpression construct, expecting to obtain FlhDC complexes containing the mutated FlhC. Unfortunately, we were not able to obtain stable and soluble FlhC protein with S31A T33A S50A mutants. We were able to obtain good protein complexes containing FlhC carrying the mutations T21A and T86A. We performed UMPylation experiments to confirm that the T21A and T86A mutants remained able to undergo UMPylation (Fig. S4A) and that the UMPylated FlhDC mutants lacked the ability to bind DNA (Fig. S4B). The above experimental results demonstrate that T21 and T86 sites are not critical sites for UMPylation and DNA-binding function and indirectly suggest that S31, T33, or S50 might be a critical site. Sequence alignment of FlhC homologues showed that S31 is highly conserved while the other sites are less conserved (Fig. S4C).

10.1128/mbio.00249-22.4 FIG S4 T21 and T86 are not essential for UMPylation-mediated FlhDC regulation. (A) *In vitro* UMPylation of FlhDC T21A and T86A. (B) EMSAs were performed for native FlhDC T21A or T86A and UMPylated FlhDC T21A or T86A with target DNA p*flhB*. (C) Sequences of FlhC from 17 different bacteria were compared using MegAlign software and the UMPylated residues T21, S31, T33, S50, and T86 are marked with inverted triangles. S31 is highly conserved across FlhC orthologs, while T21, T33, S50, and T86 are not conserved. Download FIG S4, TIF file, 2.2 MB. Copyright © 2022 Ma et al. 2022 Ma et al. https://creativecommons.org/licenses/by/4.0/ This content is distributed under the terms of the Creative Commons Attribution 4.0 International license.

### Loss of DNA-binding activity of FlhDC after UMPylation.

To determine how UMPylation modulates the function of FlhDC, the positions of UMPylation were further analyzed. Previous work reported that positively charged amino acids in positions 37 to 45 of FlhC are essential for the DNA binding activity of the FlhDC complex, indicating that this is the DNA binding region of the protein (15, 31). Structural analysis of the FlhDC complex revealed that these residues are part of the DNA binding site, which includes UMPylated sites S31 and S50 (Fig. 4C). Therefore, the UMPylation of S31 and S50 may affect the DNA binding ability of FlhDC. Residues T21 and T86 are on the inside of the ring-like structure of FlhDC, suggesting that these sites might be UMPylated before the formation of the FlhDC complex and so could not be the key sites for FlhDC regulation (Fig. S5).

10.1128/mbio.00249-22.5 FIG S5 Structural location of UMPylated sites identified in FlhC proteins. The structural presentation shows that residues S31 and S50 of FlhC are located in the DNA binding region, while T21 and T86 of FlhC are located on the inside of the ring-like structure of FlhDC. The corresponding schematic representation is shown on the right. Download FIG S5, TIF file, 1.4 MB. Copyright © 2022 Ma et al. 2022 Ma et al. https://creativecommons.org/licenses/by/4.0/ This content is distributed under the terms of the Creative Commons Attribution 4.0 International license.

**FIG 5:**
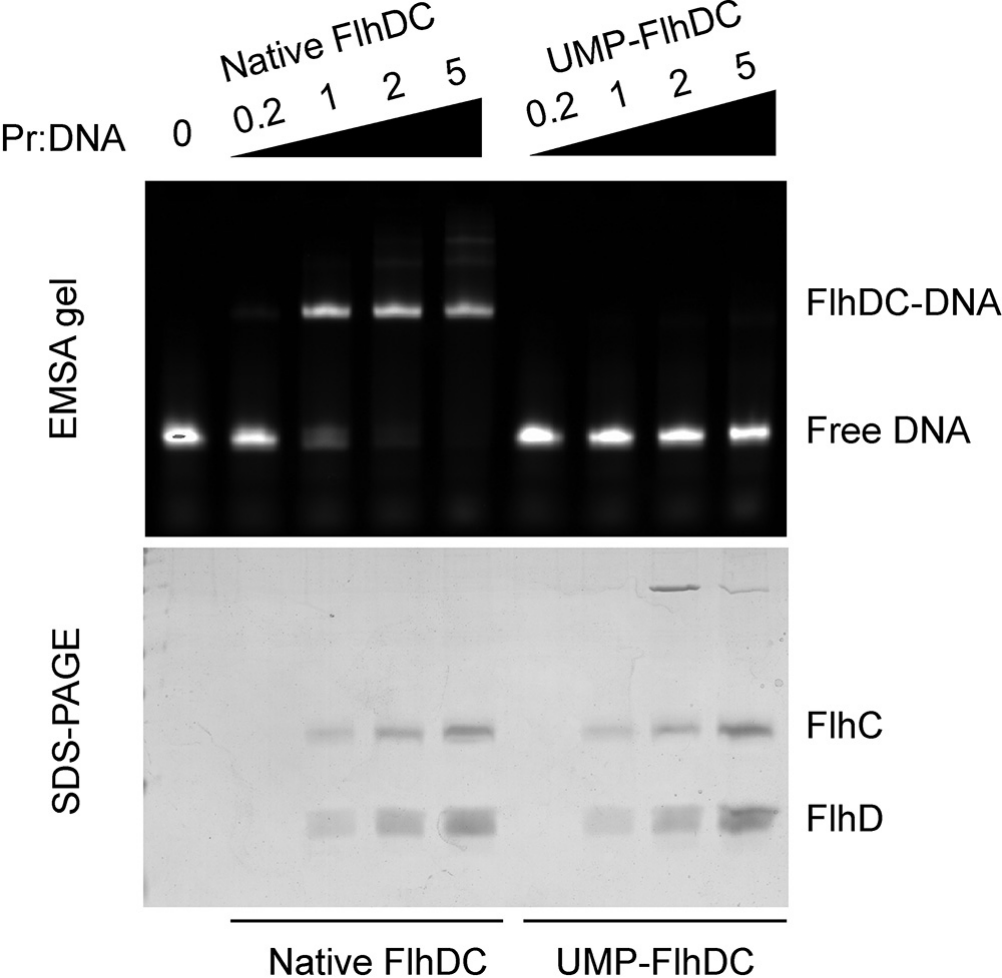
UMPylated FlhDC loses the ability to bind target DNA. EMSAs were performed for native FlhDC and UMPylated FlhDC with target DNA p*flhB* (promoter of *flhB*). In these assays, 10 nM FAM-labeled p*flhB* DNA was mixed with the indicated concentration of native FlhDC or UMPylated FlhDC for 10 min and then analyzed by EMSA. The same samples were analyzed by SDS-PAGE stained with Coomassie brilliant blue. The experiment was repeated three times, and a representative image is shown.

To investigate the influence of UMPylation on the DNA-binding activity of FlhDC, we used EMSA experiments to compare the ability of FlhDC proteins that were UMPylated *in vitro* and the native un-UMPylated FlhDC to bind a target gene. When the ratio of native FlhDC to DNA was greater than 1, native FlhDC bound to DNA and formed a complex, indicating that the purified native FlhDC had strong DNA binding capability. However, the UMPylated FlhDC failed to bind to DNA even when a protein-to-DNA ratio of 5 was used, further demonstrating that UMPylation inhibited FlhDC binding to DNA (Fig. 5). The above data indicate that the regulation of flagellar synthesis by YdiU is achieved through UMPylation and inactivation of FlhDC to restrain the transcription of downstream flagellar genes.

### YdiU helps Salmonella shut down flagellar synthesis upon entry into host cells.

**FIG 6:**
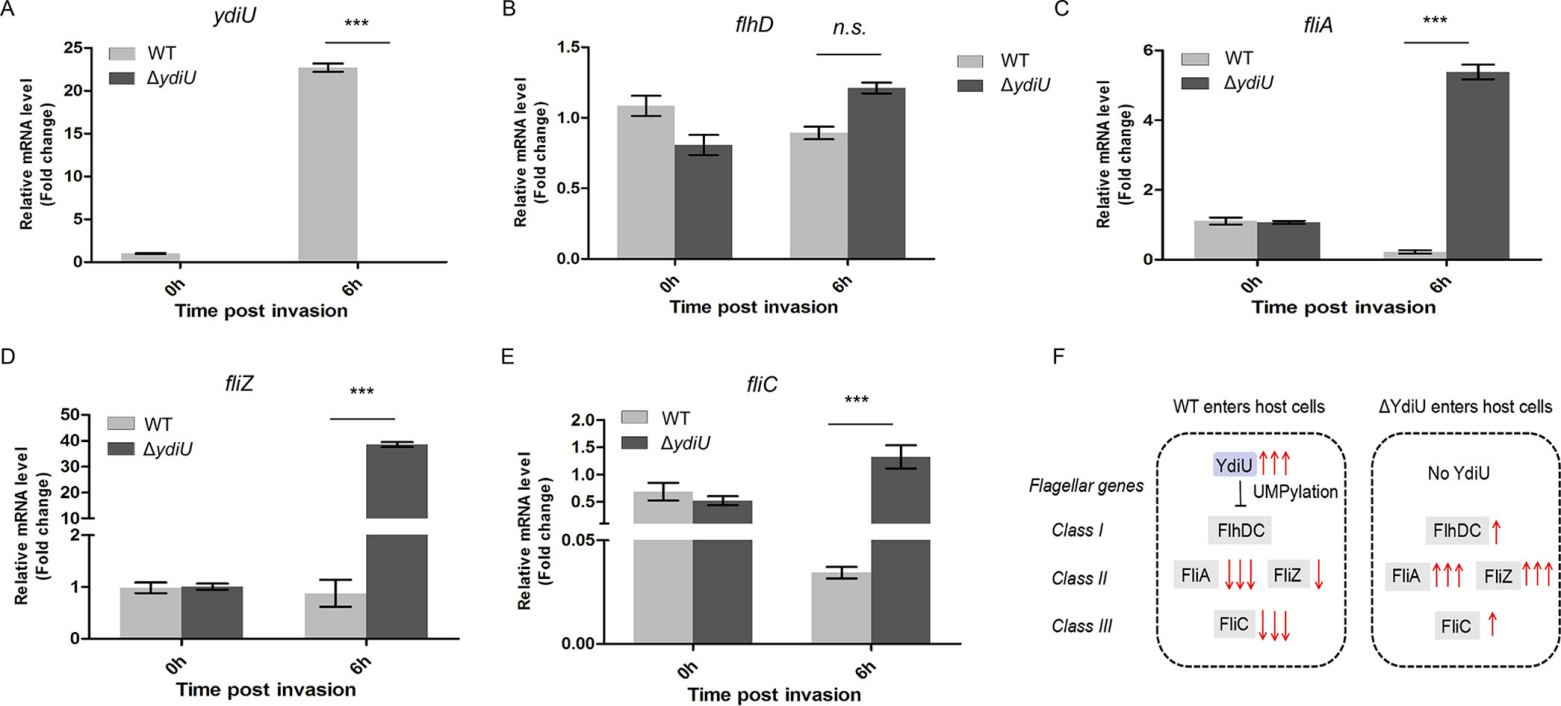
YdiU is required for flagellar control upon the entry of Salmonella into host cells. (A) The mRNA levels of *ydiU* before and 6 h after Salmonella invasion into HT-29 cells were detected in WT and Δ*ydiU* strains by qRT-PCR. (B to E) The mRNA levels of *flhD* (B), *fliA* (C), *fliZ* (D), and *fliC* (E) before and 6 h after Salmonella invasion were detected in WT and Δ*ydiU* strains by qRT-PCR. (F) Model illustrating YdiU-mediated flagellar regulation. The above-described experiments were performed as three replicates, and the means and SEM are presented. ***, *P* < 0.001; n.s., *P* > 0.05.

To further investigate the role of YdiU in Salmonella infection, wild-type and Δ*ydiU* Salmonella strains were used to infect HT-29 cells at the same multiplicity of infection. The transcriptional profiles of *ydiU* and four flagellar genes (*flhD*, *fliA*, *fliZ*, and *fliC*) before and after entry into host cells were compared by qRT-PCR (Fig. 6). After entry of the wild-type Salmonella into host cells, the expression of YdiU was significantly increased 22.7-fold, with no YdiU expression detected for the Δ*ydiU* strain (Fig. 6A). No significant change was observed in the transcriptional levels of FlhD between WT and the Δ*ydiU* strain in the host cells (Fig. 6B). Previous studies reported that the expression of the class II and class III flagellar genes quickly decreases after Salmonella enters host cells (8, 9). Consistent with this observation, our results showed that the expression levels of *fliA*, *fliZ*, and *fliC* were reduced 0.221-, 0.878-, and 0.034-fold, respectively, after WT Salmonella entered host cells (Fig. 6C to E). Instead of decreasing, the expression levels of *fliA*, *fliZ*, and *fliC* increased 5.02-, 35.90-, and 2.54-fold after the Δ*ydiU* strain entered host cells (Fig. 6C to E), indicating that YdiU was required and sufficient for flagellar control after the entry of Salmonella into host cells (Fig. 6F).

### YdiU facilitates Salmonella escape from the host immune system.

**FIG 7:**
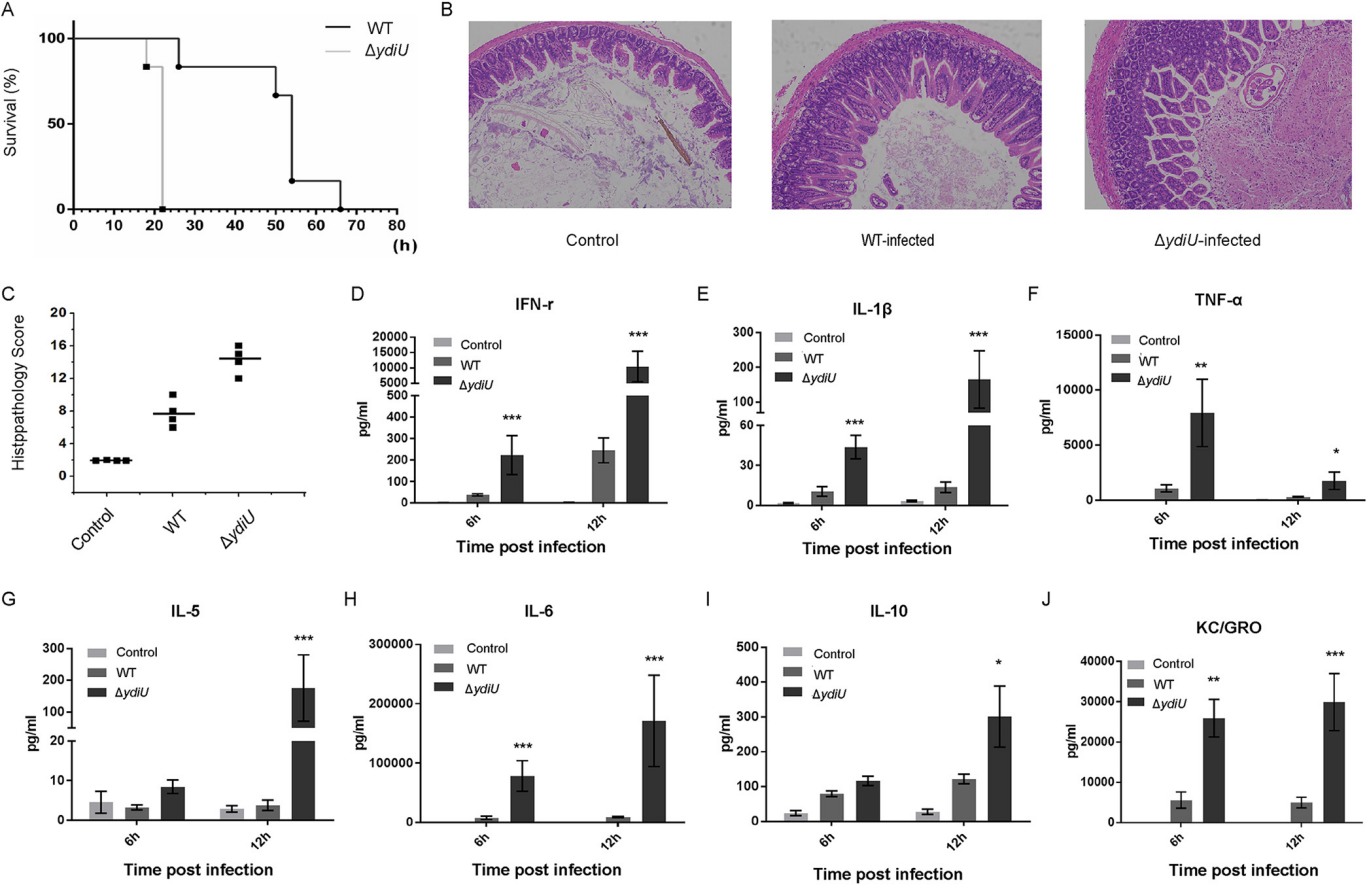
YdiU facilitates Salmonella escape from the host immune system. (A) BALB/c mice were infected with equal numbers of WT and Δ*ydiU* strains by intraperitoneal injection. Each group included five mice, and survival was monitored. (B) Pathological analysis of wild-type- and Δ*ydiU* strain-infected small intestines was conducted. Tissues were collected at 12 h postinfection (hpi). The small intestine of an uninfected mouse was used as a negative control. (C) Blind histopathology scoring of inflammation in infected small intestines showing average scores for individual animals (squares). (D to J) The levels of indicated immune factors were detected by Meso Scale Discovery (MSD) assays with the serum of mice at 6 hpi and 12 hpi. Data are from three mice per group, and means and SEM are presented. ***, *P* < 0.001; **, *P* < 0.01; *, *P* < 0.05 (compared with the wild-type strain).

To investigate the role of YdiU-mediated flagellar regulation in Salmonella pathogenesis, we next conducted animal infection experiments with BALB/c mice using both the wild-type and Δ*ydiU* strains. After intraperitoneal injection, all mice infected with the Δ*ydiU* strain died within 24 h, but the ones infected with the WT strain died only after about 3 days, revealing a significant difference in pathogenicity between wild-type and Δ*ydiU* strains (Fig. 7A). Pathological analyses of small intestines, spleens, and livers of infected mice were performed (Fig. 7B and C and Fig. S6). Blind histologic scoring of tissues showed that the mice infected wit the Δ*ydiU* strain developed severe inflammation and injury in organs compared with mice infected with wild-type Salmonella, suggesting that the rapid death of those mice might be due to immune damage. Next, serum of mice 6 h and 12 h after Salmonella infection were analyzed by Meso Scale Discovery (MSD) assays. Higher serum levels of various immune factors were observed in mice infected with the Δ*ydiU* strain than in mice infected with wild-type bacteria, with a 40-fold-higher level of interferon (IFN-γ) and a 16-fold-higher level of interleukin β (IL-β) (Fig. 7D and E). Levels of other immune factors, such as tumor necrosis factor alpha (TNF-α), KC/GRO, IL-5, IL-6, and IL-10, were also much higher in Δ*ydiU* strain-infected mice than in the wild-type-infected mice (Fig. 7F to J). All the above data demonstrated that YdiU might be an essential factor of Salmonella to mediate host immune escape.

10.1128/mbio.00249-22.6 FIG S6 Representative images of H&E-stained liver and spleen sections. Severe issue damage occurred in the spleens and livers of Δ*ydiU* strain-infected mice. Download FIG S6, TIF file, 1.2 MB. Copyright © 2022 Ma et al. 2022 Ma et al. https://creativecommons.org/licenses/by/4.0/ This content is distributed under the terms of the Creative Commons Attribution 4.0 International license.

## DISCUSSION

Flagella are required for gut colonization and pathogenicity of Salmonella (3–5, 32). Flagellin serves as a bacterial antigen that can be detected by the host pattern recognition receptors, stimulating the immune response and pyroptosis of macrophages (10, 33, 34). Pathogens have evolved strategies to shut down flagellar synthesis for immune escape, and the expression of flagellin FliC was reported to be reduced more than 10-fold upon entry into host cells (8, 9). Our group previously identified two EAL-like proteins, STM1697 and STM1344 (also known as YdiV), as factors involved in this flagellar control process (31, 35). Both STM1697 and STM1344 interact with the FlhD subunit of the FlhDC complex. Salmonella STM1697 modulates flagellar biogenesis by preventing the flagellar master protein FlhDC from recruiting RNA polymerase (35). When Salmonella lives within macrophages, STM1344 inhibits FlhDC binding to target DNA (Y. Yue, W. Wang, Y. Ma, N. Song, H. Jia, C. Li, Q. Wang, H. Li, and B. Li, under review). In this study, another factor YdiU was found to regulate flagellar biogenesis by posttranslational modification of FlhDC. To our knowledge, UMPylation is the first posttranslational modification identified for FlhDC. Obviously, posttranslational modification is a much more energy-efficient and faster way to affect FlhDC than direct protein-protein interactions, especially when Salmonella organisms are within nutrition-limited host cells.

After entry of Salmonella into host cells, the transcription of class II and class III flagellar genes was downregulated, but the transcription of *flhD* and *flhC* was significantly upregulated (8, 9). Without the coordinated regulation of STM1697, STM1344, and YdiU to alter the protein level of FlhDC, the expression of flagellin antigen will be remarkably increased after host cell entry. Our results showed that single mutation of STM1697, STM1344, or YdiU could lead to failure of Salmonella flagellar control within host cells, suggesting that the three factors were not redundant but indispensable. The different regulatory modes of these factors allow greater control to shut down flagellar production and evade host cell recognition and response.

**FIG 8:**
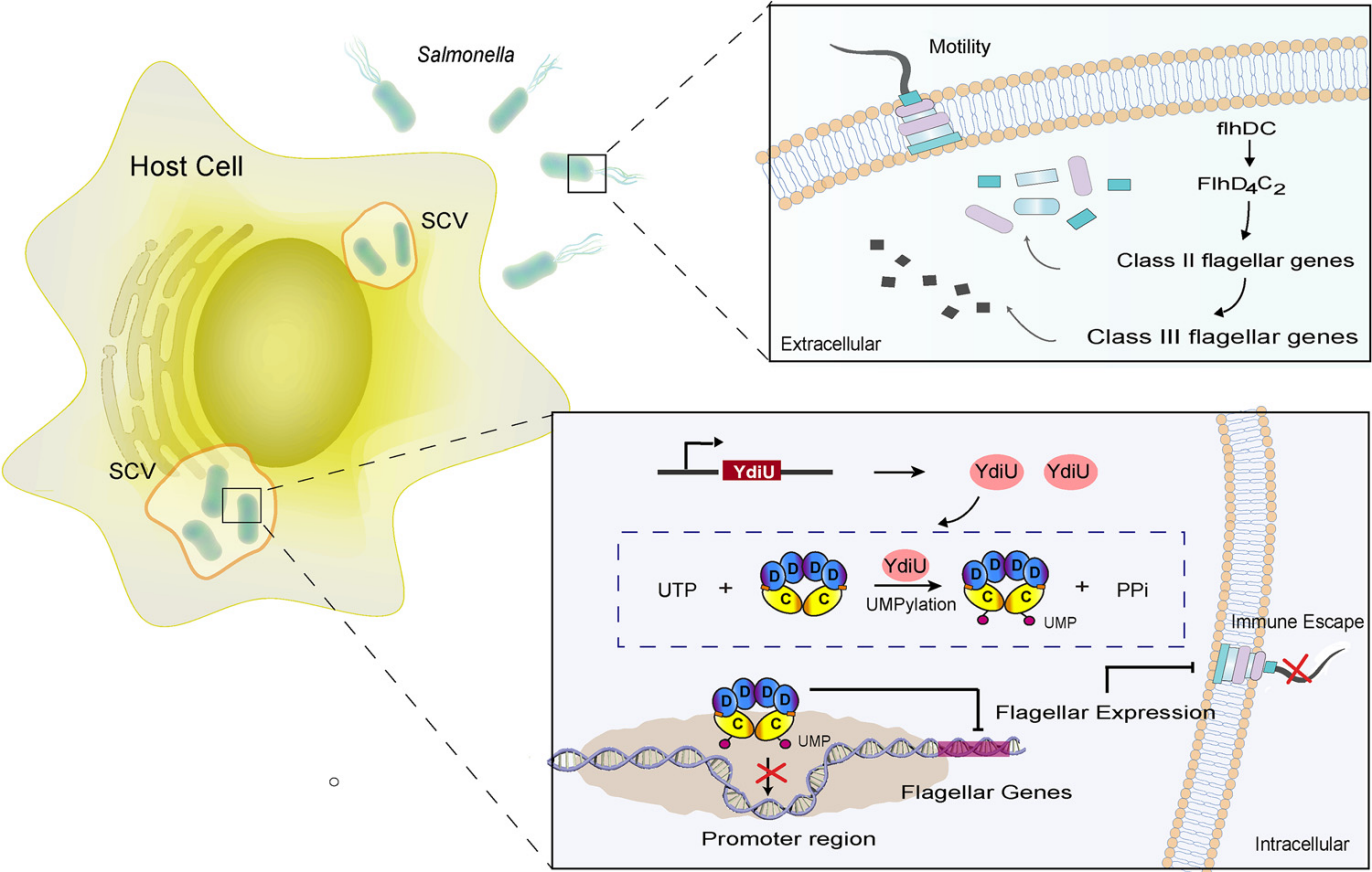
Model of YdiU-mediated flagellar regulation during Salmonella infection. When Salmonella organisms are living in an extracellular environment, unmodified FlhDC binds to the promoter region of flagellar genes and recruits RNA polymerase to promote flagellar biogenesis. After Salmonella enters host cells, YdiU is expressed and UMPylates the FlhC subunit of the FlhDC complex. UMPylated FlhDC loses the ability to bind DNA, resulting in repression of the expression of flagellar genes to shut down flagellar biogenesis. With this decrease of flagellar antigen, Salmonella can successfully escape the host immune system.

Based on these results, we constructed a model of YdiU-mediated flagellar control in Salmonella organisms that survive in the intracellular environment (Fig. 8): After Salmonella enters the host cell, the host signals, including iron deficiency, acidity, and others, stimulate the expression of YdiU. Then, a large number of YdiU proteins are expressed. YdiU UMPylates FlhDC. The UMPylated FlhDC no longer bind to the promoter region of flagellar genes. As a result, the transcription of flagellar genes and flagellar synthesis is quickly turned off. Finally, Salmonella escapes the host immune system.

Prior work suggests that YdiU UMPylates substrates at tyrosine and histidine residues, whereas serine residues were found to be important in this study (30). Although the newly identified UMPylated site exists on serine but not tyrosine/histidine residues, S31 and T33 are close to negatively charged residues (E30 and E32) and hydrophobic residues (M28, L29 and L35), which correspond to the characteristics of UMPylated peptides identified before. Given that some kinases catalyze simultaneously the phosphorylation of both Thr and Tyr, for example MEK1 (MAP2K1), we speculate that YdiU acts as a multispecific enzyme (36).

YdiU was previously identified as an UMPylator of chaperones to help Salmonella cope with ATP depletion (30). Our recent research showed that a variety of transcription factors were also UMPylated by YdiU. For example, YdiU could UMPylate the ferric uptake regulator Fur and flagellar transcription factor FlhDC *in vivo* and *in vitro*. With many negatively charged amino acids in the C-terminal regulatory domain of YdiU, YdiU may recruit transcription factors through electrostatic interaction and then UMPylate targets to achieve regulation. YdiU-mediated UMPylation might be an important global regulatory system, allowing Salmonella to adapt to an unfavorable host environment. By activating the expression of YdiU, Salmonella can initiate iron absorption, inhibit flagellar synthesis, and monitor ATP levels, potentially with other functions. Proteomic results showed that YdiU is also related to Salmonella SPI-1 virulence expression, and FlhDC and Fur may affect the expression of SPI-1 virulence (37–40). Further work is required to determine whether the regulation of virulence by YdiU is realized through FlhDC or Fur.

## MATERIALS AND METHODS

### Bacterial strains and culture conditions.

All E. coli and Salmonella enterica serovar Typhimurium strains used are listed in Table S1. In general, strains were maintained on Luria-Bertani (LB) medium or LB agar plates containing 1.5% (wt/vol) agar supplemented with antibiotics as required at 37°C. Iron-deficient medium was prepared by supplementation with 600 μM 2,2′-dipyridyl. The stock strains were activated in LB medium overnight, and then transferred to fresh medium at an optical density at 600 nm (OD_600_) of 0.01 for homogenized growth. When strains entered mid-log phase (∼OD_600_ = 0.5), 2,2′-dipyridyl and inducer were added.

10.1128/mbio.00249-22.7 TABLE S1 Strains used in this study. Download Table S1, DOCX file, 0.02 MB. Copyright © 2022 Ma et al. 2022 Ma et al. https://creativecommons.org/licenses/by/4.0/ This content is distributed under the terms of the Creative Commons Attribution 4.0 International license.

### Generation of constructs.

All plasmids and primers used in this study are listed in Tables S2 and S3. For biochemical study, *fliA* and *fliC* genes were amplified from E. coli strain K-12 substrain MG1655 genomic DNA and cloned into the pGL01 vector, and the whole *flhDC* operon was amplified from E. coli strain K-12 substrain MG1655 and cloned into the pET21b vector. FlhDC mutants (S31A, S31A/T33A, S50A, T21A, and T86A) were constructed using a site-directed mutagenesis system (TransGen Biotech, Beijing, China). *ydiU* and *flhDC* were also cloned into pUT18C and pKNT25 for the bacterial two-hybrid assay. Other plasmids were preserved in the preliminary construction of the laboratory (30, 31, 35).

10.1128/mbio.00249-22.8 TABLE S2 Plasmids used in this study. Download Table S2, DOCX file, 0.02 MB. Copyright © 2022 Ma et al. 2022 Ma et al. https://creativecommons.org/licenses/by/4.0/ This content is distributed under the terms of the Creative Commons Attribution 4.0 International license.

10.1128/mbio.00249-22.9 TABLE S3 Primers used in this study. Download Table S3, DOCX file, 0.02 MB. Copyright © 2022 Ma et al. 2022 Ma et al. https://creativecommons.org/licenses/by/4.0/ This content is distributed under the terms of the Creative Commons Attribution 4.0 International license.

### Proteomic data analysis for flagellar pathway.

Methods for mass spectrometry-based proteomics analysis are provided in another paper (45). Flagellum-associated proteins and chemotaxis-associated proteins that are expressed differentially (>2-fold or <0.5-fold) in Δ*ydiU* and wild-type Salmonella strains were selected for further analysis. Heat maps were generated using the R package pheat map. Volcano plots were generated using the R package with *P* values and relative ratios.

### RNA extraction and real-time quantitative PCR.

Total RNA was isolated from treated Salmonella using the SPARKeasy bacterial/cell RNA kit (Sparkjade, Shandong, China) according to the manufacturer’s protocol. Next, 2 μg of total RNA was used for first-strand cDNA synthesis using HiScript III RT SuperMix (Vazyme, Nanjing, China), and the cDNA produced was used as the template for quantitative real-time PCR (qRT-PCR). GapA (also known as GAPDH [glyceraldehyde-3-phosphate dehydrogenase]) was used as an internal control to normalize the different cDNA samples. The qRT-PCRs were performed using TB Green Premix Ex Taq II (TaKaRa, Japan) in the Applied Biosystems 7500 sequence detection system (Applied Biosystems). The PCR cycling conditions were as follows: 95°C for 10 min, followed by 40 cycles of 95°C for 15 s and 60°C for 60 s. The melting curve was recorded after 40 cycles to verify primer specificity by heating from 60°C to 95°C. Fold change in mRNA level was determined by the comparative cycle threshold (*C_T_*) method (41).

### Swimming motility assay.

The activated bacterial cells were inoculated at 1:100 into fresh medium, and at an OD_600_ of 0.5, dipyridine was added with a final concentration of 600 μM. After induction for 2 h, 0.6-μL bacterial suspensions were gently applied to iron-deficient 0.3% LB agar plates (supplemented with 100 g/mL ampicillin and 0.1% l-arabinose to induce YdiU expression). Each strain was assayed at least three times. Swimming motility was observed after overnight incubation at 30°C.

### Negative-stain electron microscopy.

Bacteria in the logarithmic growth phase were collected and centrifuged at 4,000 rpm for 5 min, fixed with 2.5% glutaraldehyde overnight at 4°C, and then refixed with 3% glutaraldehyde. The samples were dipped with carbon nets and dried for 5 min in a natural state. Next, samples were negatively stained with 1% phosphotungstic acid for 3 min and allowed to dry naturally. The samples were observed with a JEM-1200EX transmission electron microscope (JEOL Electronics Corporation, Japan).

### Western blotting.

Bacterial total proteins were treated at 95°C for 10 min and were separated using 12% SDS-PAGE. The proteins were then electrotransferred onto a polyvinylidene fluoride (PVDF) membrane (Millipore, Bedford, MA). The membrane was blotted with polyclonal antibodies against YdiU, FlhDC, FliC, or GapA (internal) after blocking with 5% milk in phosphate-buffered saline plus Tween (PBST) and then incubated with horseradish peroxidase (HRP)-conjugated goat anti-rabbit or -mouse IgG (Abcam) diluted in PBST. Finally, the signals were detected with chemiluminescent substrate (Immobilon Western HRP substrate; Millipore) using a FluorChem imager (UVITEC). The antibody against FliC used in this study is a polyclonal antibody purchased from Abcam (ab93713). The antibodies against YdiU, FlhDC, and GapA were prepared by Dia-An Biotech, Inc. (Wuhan, China), as described before (30, 35). For Fig. 2, the FliC levels relative to that of GapA were quantified in grayscale in three independent experiments. The gray values were gathered using ImageJ.

### Bacterial two-hybrid assay.

The bacterial adenylate cyclase-based two-hybrid (BACTH) system was used to investigate the *in vivo* interaction of YdiU and FlhDC. The BACTH assay was performed as described previously (42, 43). First, pUT18C-YdiU and pKNT25-FlhDC plasmids were constructed and then cotransformed into E. coli BTH101. To characterize the protein-protein interaction, recombinant strains were selected on LB–X-Gal (5-bromo-4-chloro-3-indolyl-β-d-galactopyranoside) plates containing 100 μg/mL ampicillin, 50 μg/mL kanamycin, 0.5 mM IPTG (isopropyl-β-d-thiogalactopyranoside), and 40 μg/mL X-Gal. To measure the strength of the interaction, β-galactosidase activity detection was carried out using a β-galactosidase activity detection kit (Solarbio, China). Positive, negative, and target strains were inoculated into LB medium containing ampicillin and kanamycin at 37°C 200 rpm and cultured to an OD_600_ of 0.5, and then IPTG was added to induce β-galactosidase gene expression. Harvested cells were lysed by sonication, and bacterial total protein was obtained to detect β-galactosidase activity.

### *In vitro* UMPylation assay.

*In vitro* UMPylation assay experiments were constructed *in vitro* by using biotin-labeled UTP as the UMP donor (biotin-16-UTP). 4 μg of purified proteins (FlhDC, FliA, or FliC) were incubated with or without 1 μg YdiU in a 10-μL reaction buffer containing 25 mM Tris-HCl (pH 7.5), 1 mM dithiothreitol (DTT), 100 mM NaCl, 10 mM MnCl_2_, and 500 mM biotin-16-UTP. After mixing of the reaction components, the reaction mixture was placed in the incubator at 30°C for 1 h and then analyzed by 12% SDS-PAGE. Then, a streptavidin-HRP blot was performed as previously described (30).

### Protein expression and purification.

The recombinant plasmids were transformed into E. coli BL21(DE3) or E. coli BL21(DE3) Δ*ydiU* for protein expression. To obtain FlhDC, FliA, and FliC without UMPylation, the E. coli BL21(DE3) Δ*ydiU* strain was used. Protein expression and purification were performed as previously described (30).

### Identification of UMPylation sites by mass spectrometry.

Four kinds of FlhDC proteins were analyzed on 12% NuPAGE gels and stained with Coomassie brilliant blue. The desired protein bands were cut from the gel and digested overnight with trypsin. The digested peptides were desalted using ZipTip C_18_ columns (Millipore, Billerica, MA), and liquid chromatography-tandem mass spectrometry (LC-MS/MS) was performed. The digestion, elution, and sampling processes were performed as previously described (30). The MS data obtained were retrieved by Mascot software in the NCBI nr database against the Escherichia coli (strain K-12) database, and UMP-modified amino acids (C_9_H_11_N_2_O_9_P; 306.025 Da) were searched for serine, threonine, tyrosine, and histidine residues. All modification sites were determined by manual retrieval mapping.

### EMSA.

As previously reported, a 49 bp *flhB* promoter in Salmonella was synthesized as the target DNA as follows (31, 35). An embedded fluorophore 6-carboxy-fluorescein (FAM)-labeled double-stranded *flhB* promoter was obtained by annealing of two complementary single-stranded FAM-*flhB* oligonucleotides. Then, the annealed DNA strands were diluted to 5 μM and preincubated with different concentrations of FlhDC (1 μM, 5 μM, 10 μM, and 25 μM) in a 20-μL reaction buffer for 10 min at 37°C. Next, samples were separated by a native 5% polyacrylamide gel in Tris-borate-EDTA (TBE) buffer at 4°C before observation using the ChemiDoc MP imaging system (Bio-Rad).

### Cell culture and bacterial infection experiments.

The human colon adenocarcinoma cell line HT-29 was cultured at 1 × 10^7^ in 100-mm tissue culture dishes with RPMI 1640 medium (Gibco) containing 10% fetal bovine serum (Gibco). Cells were seeded in 37°C cell incubator containing 5% CO_2_. Bacteria that reached the logarithmic phase were diluted with RPMI 1640 medium, and then used to infect HT-29 cells at a multiplicity of infection (MOI) of 20. After an hour of infection, the remaining extracellular bacteria were killed using RPMI 1640 with gentamicin (100 μg/mL). After that, the cells were washed twice with PBS. To keep the cells alive and inhibit extracellular bacteria, the cells were seeded with gentamicin (20 μg/mL). After infection for 6 h, cells were washed and lysed in TRIzol reagent (Tiangen) for RNA extraction. Bacteria before invasion were used as controls (0 h), and qPCR was used to compare the expression levels of flagellar genes before and after infection.

### Survival curve of BALB/c mice after infection.

Activated WT and ΔYdiU cells were inoculated at 1:100 in fresh medium and grown to an OD_600_ of 0.4. The bacterial cultures were centrifuged at 5,000 rpm for 5 min, the supernatant was discarded, and then the cells were resuspended in sterile normal saline diluted to 2 × 10^6^ cells/mL. Thirty female specific-pathogen-free (SPF) BALB/c mice (14 to 16 g) were infected with 2 × 10^5^ cells/mouse by intraperitoneal injection. The normal control group was intraperitoneally injected with 0.1 mL sterile normal saline. Animal deaths were closely monitored and mortality rates counted. All the animal experiments were approved by the animal ethics committee of the Institute of Basic Medicine, Shandong Academy of Medical Sciences, prior to their commencement.

### Pathological observation.

As described above, bacteria at the logarithmic stage were diluted with sterile normal saline to 2 × 10^6^ cells/mL, and injected intraperitoneally into 10 female SPF BALB/c mice (14 to 16 g). The control group was injected with normal saline. Sixteen hours later, the mice were sacrificed, and the small intestines, spleens, and livers were removed and fixed with tissue fixation solution before hematoxylin-and-eosin (HE) staining and microscopic observation. Blind histologic scoring of tissues was done by an experienced veterinary pathologist, as described elsewhere (44).

### Serum cytokine detection by MSD.

Blood samples were collected from BALB/c mice 6 h and 12 h after Salmonella infection. After natural coagulation, the supernatant was centrifuged at 10,000 rpm for 10 min before freezing at −80°C. The concentrations of serum cytokines were monitored with Meso Scale Discovery (MSD) assays using the proinflammatory panel 1 (mouse) V-PLEX kit according to the manufacturer’s protocol.

### Statistical analysis.

All experiments were performed with three biological replicates unless otherwise stated. A two-tailed Student's *t* test was used to calculate *P* values using SPSS. Statistical details of experiments are included in the figure legends.
