# Supplementary figures and images for: Switching off Bacterial Flagellar Biogenesis by YdiU-Mediated UMPylation of FlhDC
Source: mBio. 2022 May 9;13(3):e00249-22. doi: 10.1128/mbio.00249-22 (PMC9239255; doi:10.1128/mbio.00249-22)

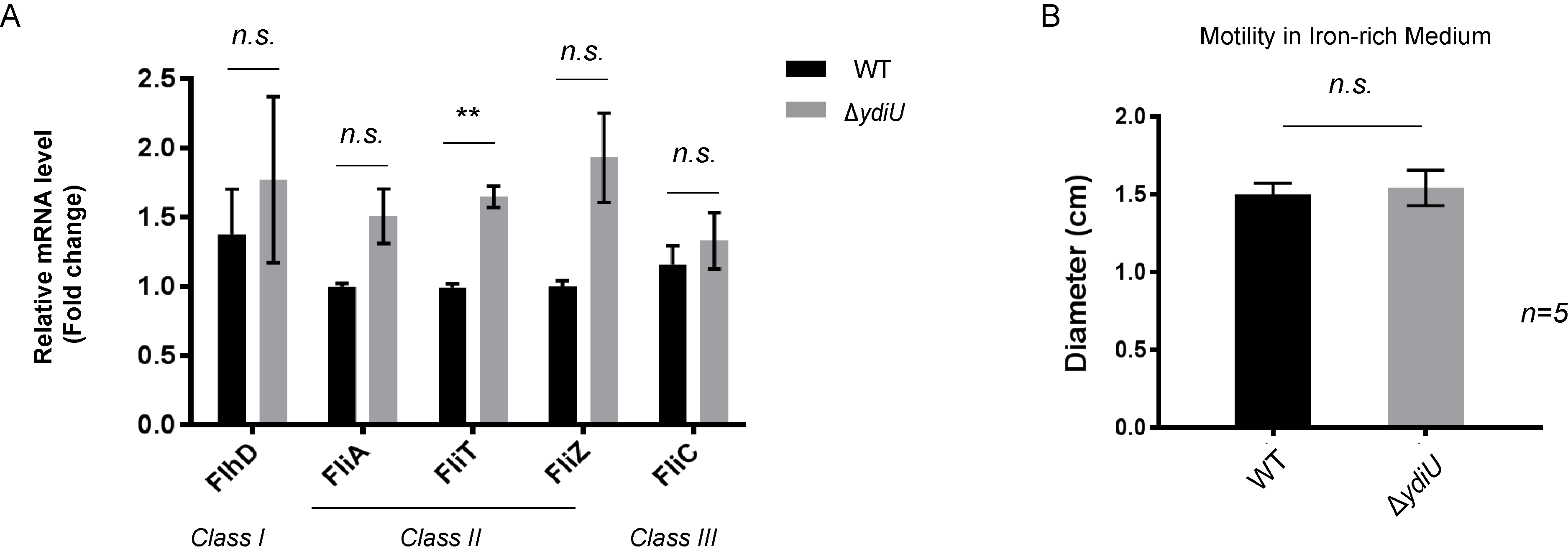

Supplement: FIG S1 [file mbio.00249-22-s0001.tif]

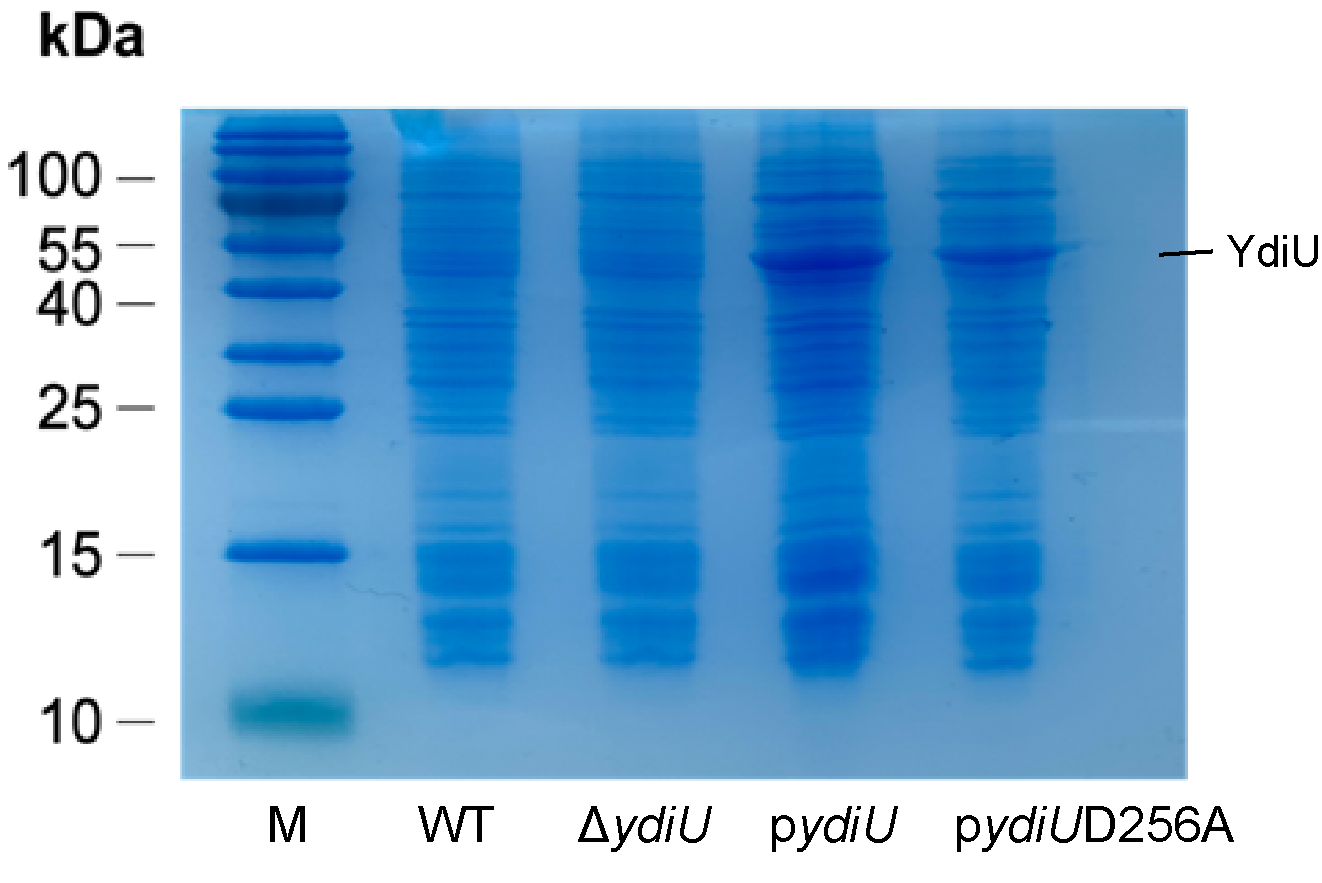

Supplement: FIG S2 [file mbio.00249-22-s0002.tif]

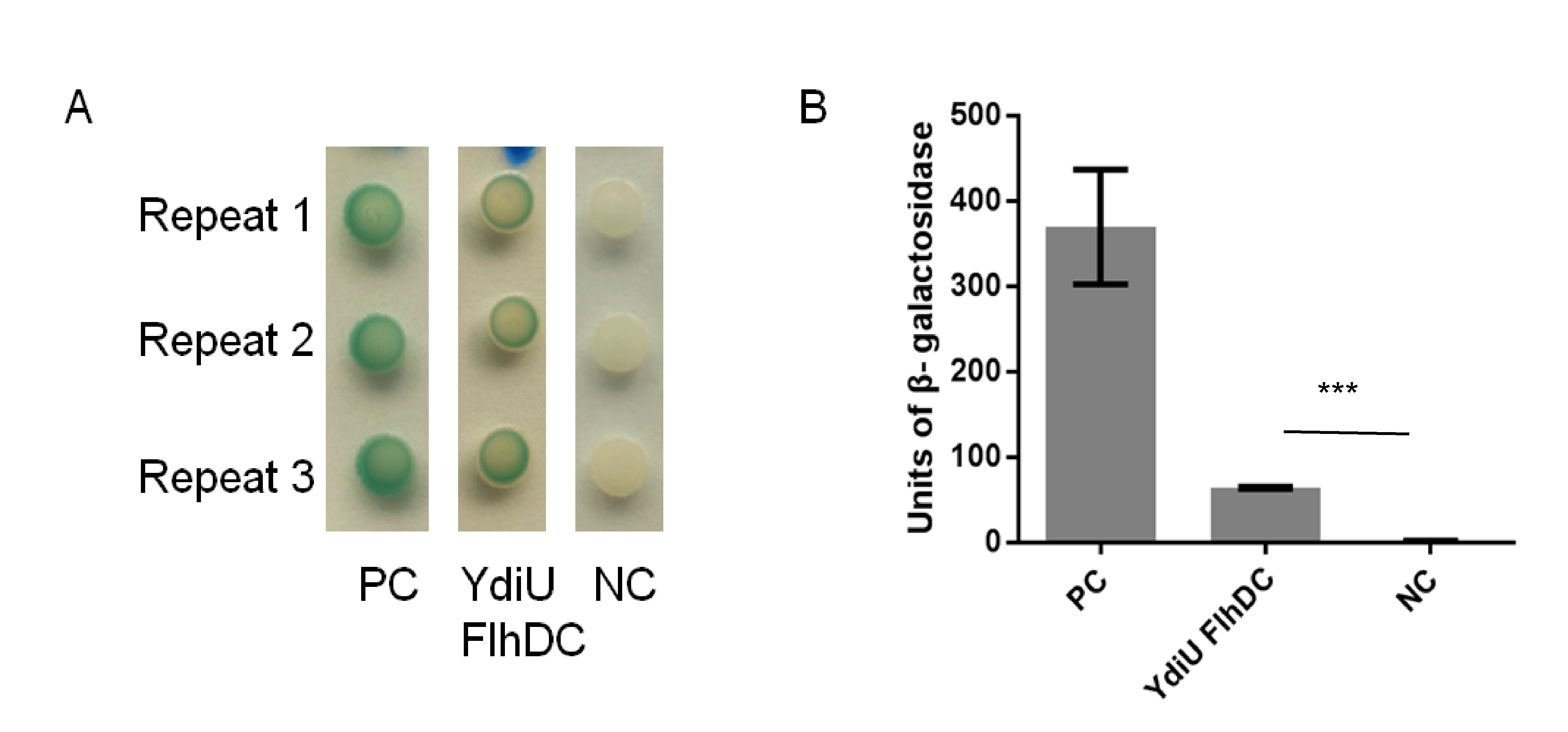

Supplement: FIG S3 [file mbio.00249-22-s0003.tif]

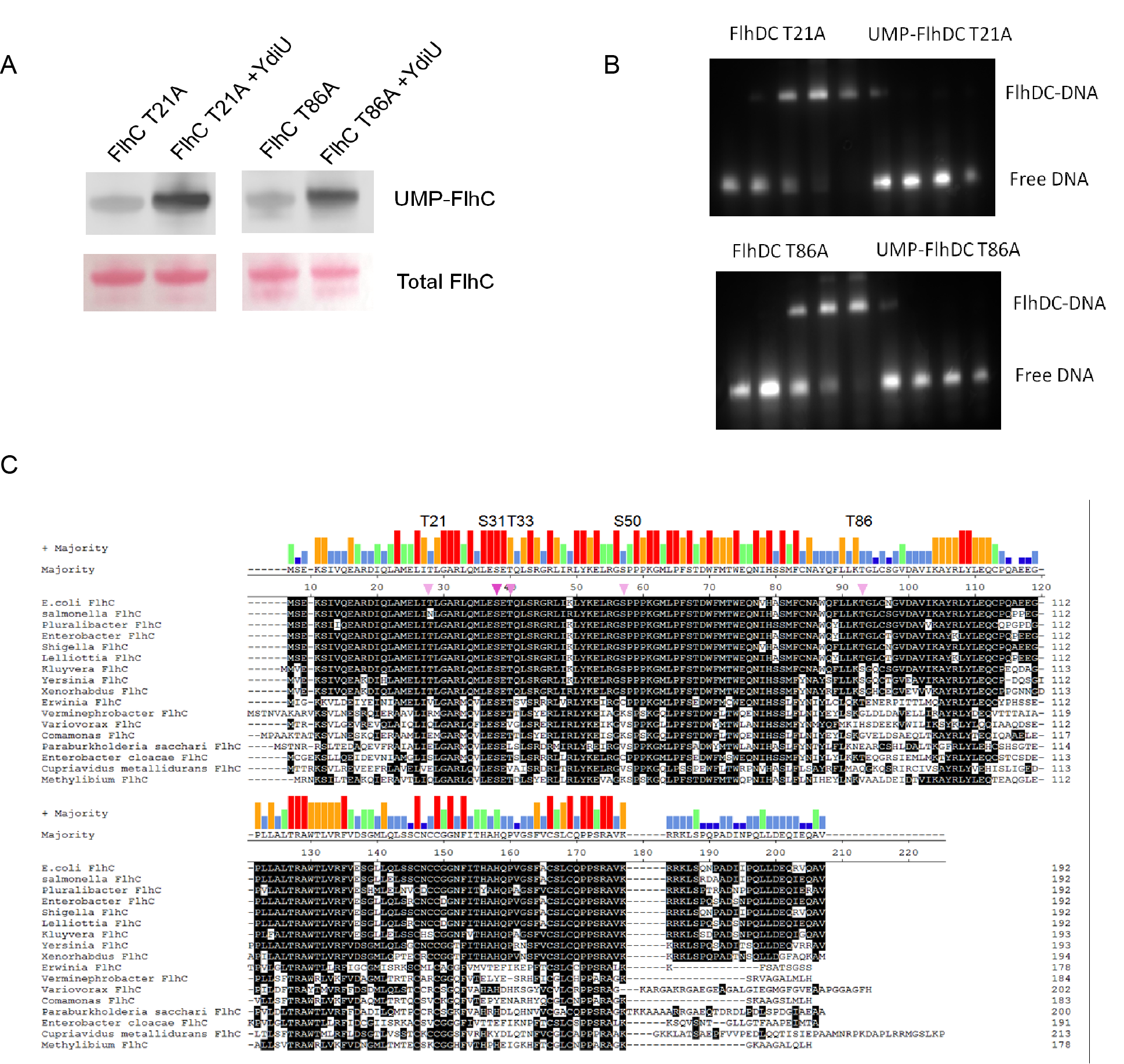

Supplement: FIG S4 [file mbio.00249-22-s0004.tif]

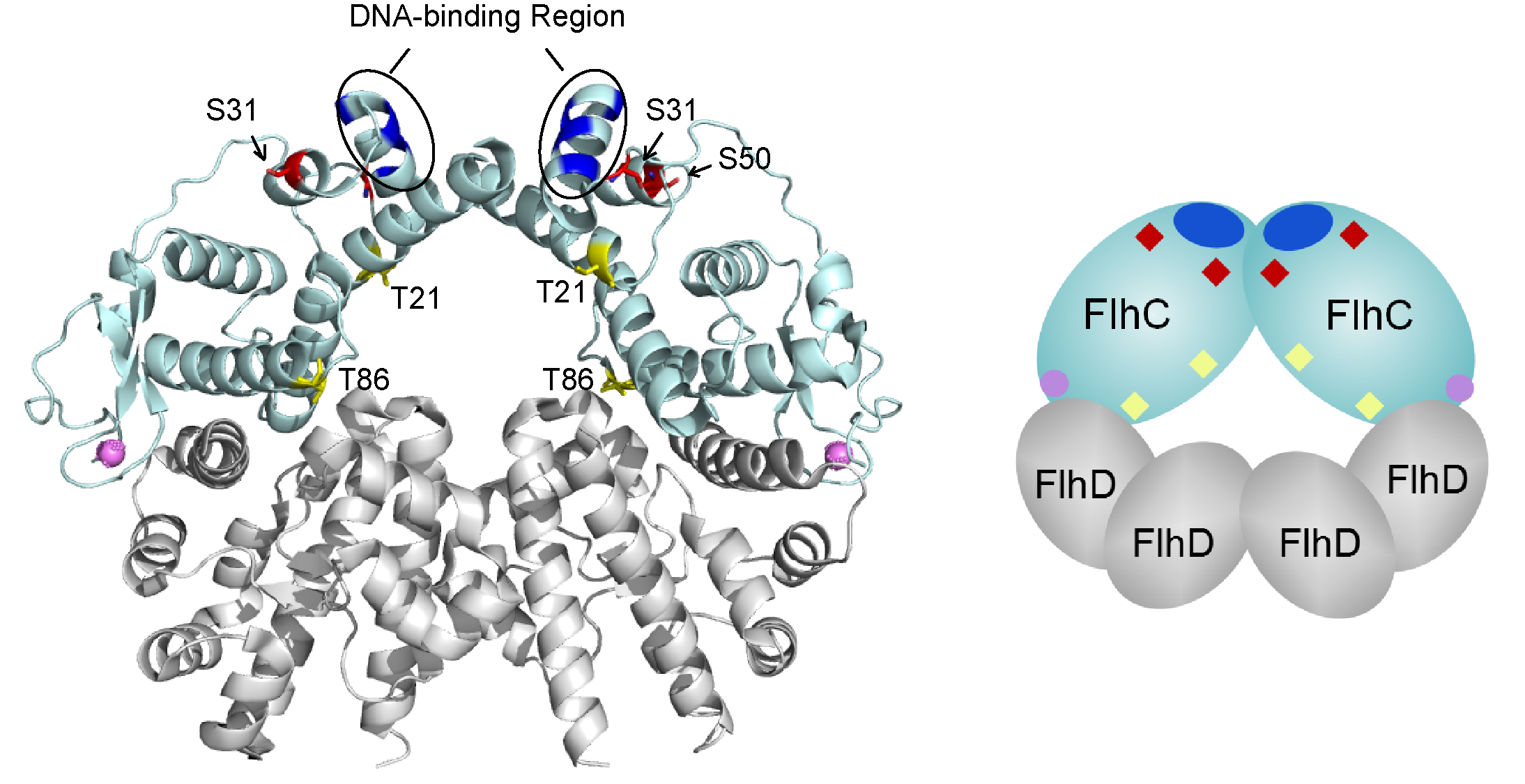

Supplement: FIG S5 [file mbio.00249-22-s0005.tif]

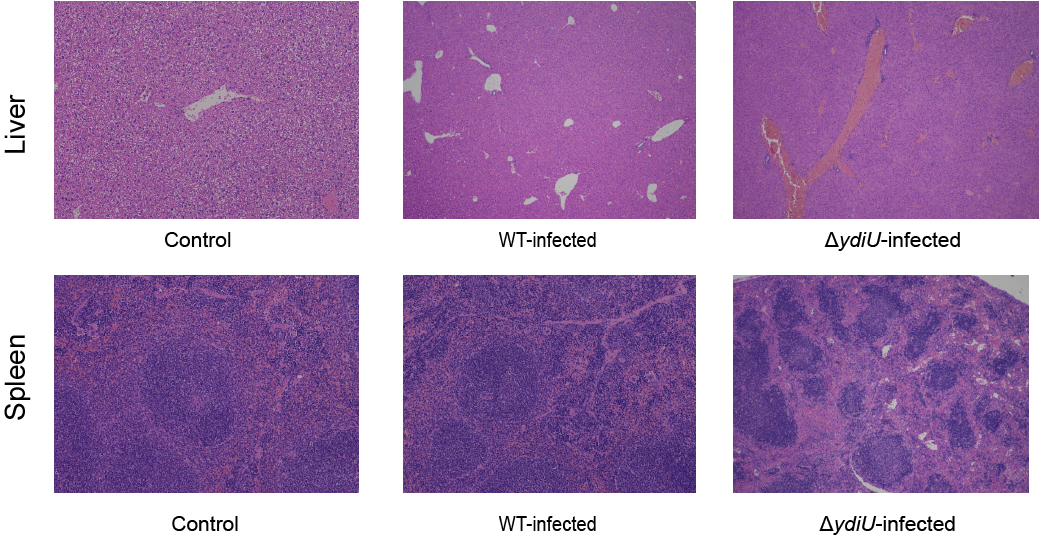

Supplement: FIG S6 [file mbio.00249-22-s0006.tif]
