# Supplementary material for: Switching off Bacterial Flagellar Biogenesis by YdiU-Mediated UMPylation of FlhDC
Source: mBio. 2022 May 9;13(3):e00249-22. doi: 10.1128/mbio.00249-22 (PMC9239255; doi:10.1128/mbio.00249-22)
Supplement: TABLE S1 [file mbio.00249-22-s0007.docx]

**Table S1. Strains used in this study**

| No | Strains | Relevant characteristic(s) | Source |
| --- | --- | --- | --- |
| 1 | WT *Salmonella* | *Salmonella enterica serovar Typhimurium* ATCC14028, no resistance | American Type Culture Collection |
| 2 | *Salmonella* Δ*ydiU* | ydiU knockout strain, no resistance | [1] |
| 3 | WT *Salmonella* VC | Vector pBad24 control, Amp^+^ | [1] |
| 4 | *Salmonella* Δ*ydiU* VC | Vector pBad24 control, Amp^+^ | [1] |
| 5 | *Salmonella* Δ*ydiU* p*ydiU* | YdiU^475^/pBad24, Amp^+^ | [1] |
| 6 | *Salmonella*Δ*ydiU*p*ydiU* D256A | YdiU^475^D256A/pBad24, Amp^+^ | [1] |
| 7 | *E. coli* BL21(DE3) | T7 expression host, no resistance | Takara Bio Inc. |
| 8 | *E. coli* BL21(DE3)Δ*ydiU* | T7 expression host, no resistance | [1] |
| 9 | *E. coli* BL21(DE3)p*flhDC* | FlhDC/pET21b, Amp^+^ | This study |
| 10 | *E. coli* BL21(DE3)p*fliA* | FliA/pGl01, Amp^+^ | This study |
| 11 | *E. coli* BL21(DE3)p*fliC* | FliC/ pGl01, Amp^+^ | This study |
| 12 | *E. coli* BL21(DE3)p*ydiU* | YdiU^475^/ pGl01, Amp^+^ | [1] |
| 13 | *E. coli* BL21(DE3)p*ydiU*29b | YdiU^475^/ pET29b (no tag), Kan^+^ | [1] |
| 14 | *E. coli* BL21(DE3)p*ydiU* pFlhDC | FlhDC/pET21b, Amp^+^  YdiU^475^/ pET29b (no tag), Kan^+^ | This study |
| 15 | *E. coli* BL21(DE3)ΔYdiU pFlhDC | Δ*ydiU* FlhDC/pET21b, Amp^+^ | This study |
| 16 | *E. coli* BTH101 | no resistance , strain used for bacterial two-hybrid assay | [2] |
| 17 | *E. coli* BTH101-PC | zip/pKNT25, Kan^+^; zip/pUT18C, Amp^+^, Positive control for bacterial two-hybrid assay | [2] |
| 18 | *E. coli* BTH101-NC | Vector pKNT25 control Kan^+^, Vector pUT18C control Amp^+,^ Negative control for bacterial two-hybrid assay | [2] |
| 19 | *E. coli* BTH101-FlhDC- YdiU | FlhDC/pKNT25, Kan^+^; YdiU/pUT18C, Amp^+^ | This study |

[1] Yang, Y., Yue, Y., Song, N., Li, C., Yuan, Z., Wang, Y., ... & Li, B. (2020). The YdiU domain modulates bacterial stress signaling through Mn^2+^-dependent UMPylation. *Cell Reports*, 32(12), 108161.

[2] Zhang, F., Li, B., Dong, H., Chen, M., Yao, S., Li, J., ... & Gu, L. (2020). YdiV regulates Escherichia coli ferric uptake by manipulating the DNA-binding ability of Fur in a SlyD-dependent manner. *Nucleic acids research*, 48(17), 9571-9588.
