# Supplementary material for: Switching off Bacterial Flagellar Biogenesis by YdiU-Mediated UMPylation of FlhDC
Source: mBio. 2022 May 9;13(3):e00249-22. doi: 10.1128/mbio.00249-22 (PMC9239255; doi:10.1128/mbio.00249-22)
Supplement: TABLE S2 [file mbio.00249-22-s0008.docx]

**Table S2. Plasmids used in this study**

| No | Plasmids | Relevant characteristic(s) | Source |
| --- | --- | --- | --- |
| 1 | pBad24 | Vector pBad24 control Amp^+^ | [1] |
| 2 | YdiU^475^/pBad24 | *Salmonella* YdiU^1-475aa^ cloned into pBad24 | [2] |
| 3 | YdiU^475^D256A/pBad24 | *Salmonella* YdiU^1-475aa^ D256A cloned into pBad24 | [2] |
| 4 | pGL01 | Expression Vector Amp^+^ | [3] |
| 5 | YdiU^475^/pGL01 | YdiU 1-475aa cloned into pGL01 | [2] |
| 6 | YdiU^475^/pET29b | YdiU 1-475aa cloned into pET29b | [2] |
| 6 | FlhDC/pET21b | FlhDC operon cloned into pET21b | This study |
| 7 | FliA /pGL01 | FliA full-length cloned into pGL01 | This study |
| 8 | FliC /pGL01 | FliC full-length cloned into pGL01 | This study |
| 9 | pET29b | Expression Vector Kan^+^ | Novagen |
| 10 | YdiU^475^/pET29b(3T) | YdiU 1-475aa cloned into pET29b | [2] |
| 11 | FlhDC /pKNT25 | FlhDC full-length cloned into pKNT25 | This study |
| 12 | YdiU /pUT18C | YdiU full-length cloned into pUT18C | This study |

[1] Guzman, L. M., Belin, D., Carson, M. J., & Beckwith, J. O. N. (1995). Tight regulation, modulation, and high-level expression by vectors containing the arabinose PBAD promoter. *Journal of bacteriology*, 177(14), 4121-4130.

[2] Yang, Y., Yue, Y., Song, N., Li, C., Yuan, Z., Wang, Y., ... & Li, B. (2020). The YdiU domain modulates bacterial stress signaling through Mn^2+^-dependent UMPylation. *Cell Reports*, 32(12), 108161.

[3] Li, B., Li, N., Wang, F., Guo, L., Huang, Y., Liu, X., ... & Gu, L. (2012). Structural insight of a concentration-dependent mechanism by which YdiV inhibits *Escherichia coli* flagellum biogenesis and motility. *Nucleic acids research*, 40(21), 11073-11085.
