# Supplementary material for: Switching off Bacterial Flagellar Biogenesis by YdiU-Mediated UMPylation of FlhDC
Source: mBio. 2022 May 9;13(3):e00249-22. doi: 10.1128/mbio.00249-22 (PMC9239255; doi:10.1128/mbio.00249-22)
Supplement: TABLE S3 [file mbio.00249-22-s0009.docx]

**Table S3. Primers used in this study**

| No | Oligonucleotide | Sequence | Application |
| --- | --- | --- | --- |
| 1 | flhDC-21b-F | ATACATATGCATACCTCCGAGTTGCTG | Gene Clone |
| 2 | flhDC-21b-R | ATACTCGAGTTAAACAGCCTGTACTCTCTGTTC | Gene Clone |
| 3 | flhDC-T25-F | ATATCTAGAatgcatacctccgagttgc | Gene Clone |
| 4 | flhDC-T25-R | ATAGGTACCAGCCTGTACTCTCTGTTCATC | Gene Clone |
| 5 | ydiU-T18-F | ATATCTAGAATGACCCTGTCTTTTACTGCC | Gene Clone |
| 6 | ydiU-T18-R | ATAGGTACCACTTGAACAACTGACCTCCAG | Gene Clone |
| 7 | fliA-pGl01-F | ATAGGATCCGTGAATTCACTCTATACCGCT | Gene Clone |
| 8 | fliA-pGl01-R | ATACTCGAGTTA TAACTTACCCAGTTTAGTGCG | Gene Clone |
| 9 | fliC-pGl01-F | ATAGGATCCATGGCACAAGTCATTAATAC | Gene Clone |
| 10 | fliC-pGl01-R | ATACTCGAGTTAACCCTGCAGCAGAGACAGAA | Gene Clone |
| 11 | flhDC-S31A-F | ctgggcgctcgtttgcagatgctggaaGCAgaatcacagttaagtcgcggacgcctgataaaact | Gene Clone |
| 12 | flhDC-S31A-R | agttttatcaggcgtccgcgacttaactgtcattcTGCttccagcatctgcaaacgagcgcccag | Gene Clone |
| 13 | flhDC-S31AT33A-F | ctgggcgctcgtttgcagatgctggaaGCAgaaGCAcagttaagtcgcggacgcctgataaaact | Gene Clone |
| 14 | flhDC-S31AT33A-R | agttttatcaggcgtccgcgacttaactgTGCttcTGCttccagcatctgcaaacgagcgcccag | Gene Clone |
| 15 | flhDC-S50A-F | ctgataaaactttataaagaactgcgcggaGCAccaccgccgaaaggcatgctgccatt | Gene Clone |
| 16 | flhDC-S50A-R | aatggcagcatgcctttcggcggtggTGCtccgcgcagttctttataaagttttatcag | Gene Clone |
| 17 | flhDC-T21A-F | gcgggatattcagctggcaatggaattgatcGCActgggcgctcgtttgcagatgctggaa | Gene Clone |
| 18 | flhDC- T21A-R | ttccagcatctgcaaacgagcgcccagTGCgatcaattccattgccagctgaatatcccgc | Gene Clone |
| 19 | flhDC- T86A-F | gttctgtaatgcatggcagtttttactgaaaGCAggtttgtgtaatggcgtcgatgcggtgat | Gene Clone |
| 20 | flhDC- T86A-R | atcaccgcatcgacgccattacacaaaccTGCtttcagtaaaaactgccatgcattacagaac | Gene Clone |
| 21 | ydiU-5 | aactggcgcagcagttag | qPCR |
| 22 | ydiU-3 | gatgcccgctatatacctg | qPCR |
| 23 | flhD-5 | CGCCTCGGTATCAACGAAGA | qPCR |
| 24 | flhD-3 | CTCCGCCAGTTTGACCATCT | qPCR |
| 25 | fliA-5 | cttacccagtttggtgcg | qPCR |
| 26 | fliA-3 | cgagcaactggtgttaac | qPCR |
| 27 | fliT-5 | AAACTCCACCGGGCATTACG | qPCR |
| 28 | fliT-3 | TGCTCATTGTCCAGCGTTTG | qPCR |
| 29 | fliZ-5 | cagaactggcggtaaaggg | qPCR |
| 30 | fliZ-3 | catttcccacgatctgctgc | qPCR |
| 31 | fliC-5 | cgcagtaaagagaggacg | qPCR |
| 32 | fliC-3 | gggcaacaccgtaaacaacc | qPCR |
| 33 | gapA-5 | gaccttcgatgatgccgaag | qPCR |
| 34 | gapA-3 | gccaggacatcgtttccaac | qPCR |
| 35 | ﬂhB promoter-5 | FAM-ATTAACGCCATAAACCCCGCCTTTTTTACC GCTTACTCTGCCTATTGGCGT | EMSA |
| 36 | ﬂhB promoter-3 | ACGCCAATAGGCAGAGTAAGCGGTAAAAAAGGC GGGGTTTATGGCGTTAAT | EMSA |
